# Extracellular Vesicles: Potential Role in Remote Signaling and Inflammation in *Trypanosoma cruzi*-Triggered Disease

**DOI:** 10.3389/fcell.2021.798054

**Published:** 2021-12-20

**Authors:** Luíza Dantas-Pereira, Rubem Menna-Barreto, Joseli Lannes-Vieira

**Affiliations:** ^1^ Laboratório de Biologia Celular, Instituto Oswaldo Cruz, Fundação Oswaldo Cruz, Rio de Janeiro, Brazil; ^2^ Laboratório de Biologia das Interações, Instituto Oswaldo Cruz, Fundação Oswaldo Cruz, Rio de Janeiro, Brazil

**Keywords:** *Trypanosoma cruzi*, Chagas disease, extracellular vesicles, inflammation, immune response

## Abstract

Extracellular vesicles (EVs) act as cell communicators and immune response modulators and may be employed as disease biomarkers and drug delivery systems. In infectious diseases, EVs can be released by the pathogen itself or by the host cells (infected or uninfected), potentially impacting the outcome of the immune response and pathological processes. Chagas disease (CD) is caused by infection by the protozoan *Trypanosoma cruzi* and is the main cause of heart failure in endemic areas. This illness attracted worldwide attention due to the presence of symptomatic seropositive subjects in North America, Asia, Oceania, and Europe. In the acute phase of infection, nonspecific signs, and symptoms contribute to miss diagnosis and early etiological treatment. In this phase, the immune response is crucial for parasite control; however, parasite persistence, dysregulated immune response, and intrinsic tissue factors may contribute to the pathogenesis of chronic CD. Most seropositive subjects remain in the indeterminate chronic form, and from 30 to 40% of the subjects develop cardiac, digestive, or cardio-digestive manifestations. Identification of EVs containing *T. cruzi* antigens suggests that these vesicles may target host cells and regulate cellular processes and the immune response by molecular mechanisms that remain to be determined. Parasite-released EVs modulate the host-parasite interplay, stimulate intracellular parasite differentiation and survival, and promote a regulatory cytokine profile in experimental models of CD. EVs derived from the parasite-cell interaction inhibit complement-mediated parasite lysis, allowing evasion. EVs released by *T. cruzi*-infected cells also regulate surrounding cells, maintaining a proinflammatory profile. After a brief review of the basic features of EVs, the present study focuses on potential participation of *T. cruzi*-secreted EVs in cell infection and persistence of low-grade parasite load in the chronic phase of infection. We also discuss the role of EVs in shaping the host immune response and in pathogenesis and progression of CD.

## General Overview

Despite well-recognized types of cell-to-cell communication, extracellular vesicles (EVs) have emerged in the past 3 decades as an important strategy for delivery of cargo to short or long distances between the cells, acting through EV uptake or receptor-mediated interactions (van Niel et al., 2018). This cell communication process occurs during physiological and pathological processes between healthy cells and microorganisms and is preserved in evolution (Woith et al., 2019). The relevance of EVs is high; thus, the International Society for Extracellular Vesicles was created in 2012 to stimulate and promote the advances in this field of research, organize scientific meetings, and standardize the protocols (Théry et al., 2018). Recent EV studies suggested their biomedical uses, including biomarkers of diseases and possible delivery of drugs and modulators of immune response, topics previously revised (Latifkar et al., 2019; Luo et al., 2021). The present review initially provides a general overview of the field to introduce the subject and emphasize important and common topics, which will assist with subsequent focus on the main goal, i.e., the release and action of EVs in a scenario of infection by the protozoan parasite *Trypanosoma cruzi*.

## Extracellular Vesicles: An Introduction

EVs can be released by all cell types and are composed of a non-replicant lipid bilayer containing nucleic acids (DNA, RNA, and miRNA), proteins and lipids (van Niel et al., 2018). To date, two subtypes of EVs have been described based on their size and biogenesis. The vesicles derived from the plasma membrane are known as microvesicles (referred to as ectosomes, shedding vesicles, or large EVs), with size distribution from 50 nm to 1 µm depending on the cell type (Stein and Luzio, 1991). Endosome-borne vesicles are derived from the formation of a multivesicular body (MVB), which is filled with intraluminal vesicles that fuse with the plasma membrane, and are known as exosomes (or small EVs) with a size ranging from 50∼150 nm (Théry, 2011). Unfortunately, there is no consensus on EV markers applicable to all cell systems, which can distinguish both types of EVs; thus, EV tags vary based on the progenitor cell type and experimental system (Théry et al., 2018). In the present review, we describe proposed biogenesis of small and large vesicles; however, no distinction will be made between these vesicles, which are collectively named EVs in the text.

The release of EVs is a conserved process in living beings. MVBs were described for the first time in algae and have been identified in archaea, bacteria, fungi, and mammalian cells (Sager and Palade, 1957; Chatterjee et al., 1959; Sotelo and Porter, 1959; Takeo et al., 1973; Deatherage and Cookson, 2012). Secretion of the particles as a result of the fusion of MVBs with the plasma membrane of rat reticulocytes was described in 1987, and the term “exosomes” was used for the first time (Johnstone et al., 1987). Nonetheless, potential functions of EVs remained unclear, and the biological roles of EVs were initially unveiled when exosomes released by B lymphocytes and dendritic cells were shown to act as immune regulators, stimulating T cell proliferation and suppressing tumor growth in a T cell-dependent manner (Raposo et al., 1996; Zitvogel et al., 1998). Additionally, the microvesicles derived from monocytes were reported to be involved in blood coagulation (Satta et al., 1994). These findings suggested that EVs may play a role in physiological functions and also act in pathophysiological events, paving the way for an entirely new field of research.

To date, EVs are known to be secreted by some prokaryotic organisms and virtually all types of eukaryotic cells. Furthermore, EVs have been detected in cell culture-conditioned media, dissociated tissue, and biofluids, such as cerebrospinal fluid, aqueous humor, nasal secretions, saliva, bronchoalveolar lavage, pleural effusions, breast milk, ascitic fluid, bile, amniotic fluid, urine, synovial fluid, and plasma; thus, EVs are a potentially widespread communication tool in a complex organism (Witwer et al., 2013). Moreover, EVs act as interkingdom crosstalk communicators in an invader-host interplay, modulating the immune response and changing gene expression in receiver cells, e.g., via RNA delivery (Tsatsaronis et al., 2018). In recent decades, a wide range of functions of EVs have been described in several infectious diseases and pathological conditions. Most of the publication on EV field are in cancer research, contributing to shed light on crucial biological processes. For instance, EVs derived from tumor cells form pre-metastatic niches in diverse organs, enhance tumor immune escape and may be involved in drug resistance transfer between cells, contributing to tumor progression (Dong et al., 2021). Conversely, there is the perspective of usage of tumor shed EVs as biomarkers of the diagnosis and/or prognosis of a variety of cancer types, as well as therapeutic targets to prevent chemoresistance of cancer cells (Zhong et al., 2021). The present review discusses some points that may contribute to the potential role of EVs in remote signaling and inflammation in infection and tissue injury.

## Biogenesis of Extracellular Vesicles

### Exosomes/Small EVs

Almost all studies on ectosome and exosome biosynthesis, including mechanistic theories, are related to mammalian cells, and very little is known about the process in protozoans. EV cargo is influenced by the cellular physiological state and site of the production, either toward the plasma membrane (microvesicles or large EVs) or toward the MVBs (exosomes or small EVs). Even though the sites of origin of EVs are different, biosynthetic pathways of both EVs share common mechanisms and sorting machinery.

Exosomes are formed from the endocytic pathway, where an invagination of the plasma membrane leads to the formation of an early endosome, which may fuse with the vesicles originating from the Golgi, generating late endosomes. Invagination of the late endosome membranes generates the vesicles inside the lumen, known as intraluminal vesicles (ILVs); these endosomes containing ILVs are known as MVBs. This process is promoted by the endosome sorting complexes required for transport (ESCRT) machinery present on the MVB membrane, which is comprised of ESCRT subcomplexes, ATPases, and accessory proteins (van Niel et al., 2018). Recruitment of ESCRT is triggered by phosphatidylinositol-3-phosphate (PIP3), which recruits the subcomplexes ESCRT-0, ESCRT-I, and clathrin, clustering the ubiquitinated transmembrane proteins on the microdomains. The subunit ESCRT-III promotes budding and fission of the vesicles inwards via ESCRT-II. Alternatively, various combinations of accessory proteins, such as Alix and HD-PDP, syntenin, syndecans, protease-activated receptor-1, and ESCRT 0, I, and III, can lead to the formation of MVB and cargo sorting (Baietti et al., 2012; Teng and Fussenegger, 2020). Inactivation of the ESCRT components influences the efficiency of the secretion and composition of secreted vesicles, enabling active selection of exosome subpopulations by ESCRT (Colombo et al., 2013).

Alternative mechanisms of MVB formation independent of the ESCRT machinery have been described in oligodendroglial cells, where the lipid ceramide promotes negative curvature of the endosomal membrane, leading to ILV formation, suggesting that exosome formation mechanisms are variable based on cargo diversity (Trajkovic et al., 2008). Additionally, tetraspanin family proteins, such as CD63, CD81, CD82, and CD9, have been associated with cargo recruitment, forming clusters in the MVB membrane with other tetraspanins, cytosolic proteins, and transmembrane proteins and creating microdomains that bud into the MVBs (van Niel et al., 2011). Recently, the release of nonexosomal vesicles (endosome-derived vesicles without specific exosome markers or originating from the plasma membrane) was reported to be facilitated by N-glycosylation in melanoma cells. Inhibition of N-glycosylation did not impair exosomal secretion associated with tetraspanins in these cells, suggesting an additional mechanism of EV biogenesis that deserves further study (Harada et al., 2020).

MVB formation may lead to two possible outcomes: 1) MVB fusion with lysosomes/autophagosomes or 2) MVB fusion with the plasma membrane, enabling the release of ILVs to the extracellular medium as the exosomes. Recently, balance between the degradation and secretion of MVBs was assessed; however, the studies remain in progress. Atg12 and 13 are the ubiquitin-like proteins required for early steps of autophagy and interact with Alix (an accessory protein of exosome biogenesis) to promote exosome biogenesis (Murrow et al., 2015). Another possible mechanism crucial for homeostasis was described in astrocytes; the mechanism involves an association of prion protein (PNRP) with caveolin-1, which suppresses autophagy by avoiding degradation of the MVB content to favor exosome release (Dias et al., 2016a). Ubiquitin-like modification of TSG101, which is a protein component of the ESCRT-I machinery, was demonstrated to promote its aggregation and degradation, impairing exosome secretion (Villarroya-Beltri et al., 2016). MVB transport to the cell periphery is mediated by small GTPases, such as Rab11, Rab35, and Rab27a, involving cytoskeletal elements, motor molecules, and Ca^++^ mobilization (Blanc and Vidal, 2018). MVB fusion to the plasma membrane is mediated by actin, SNAP23, and soluble NSF attachment receptor (SNARE) proteins, such as VAMP3, VAMP7, and syntaxin 1A, to form a complex binding site for the membrane fusion machinery (Teng and Fussenegger, 2020).

All proteins associated with ILV biogenesis are also involved in cargo sorting of the exosomes. In addition, cytosolic proteins participate in protein and RNA sorting (such as HSP70 and RNA-binding proteins) once exosomes emerge from the endosomal compartment. A number of the exosome membrane components associated with MVB formation, membrane transport, and integrin-mediated adhesion have already been identified, including tetraspanins, major histocompatibility complex (MHC)-mediated antigen presentation, various nucleic acids (distinct RNAs and DNAs), lipids (ceramide, phosphatidylserine, and cholesterol), lipid rafts (especially rafts related to flotillin), polysaccharides, and glycans (Kalra et al., 2016).

### Microvesicles/Large EVs

Identification of the mechanisms of microvesicle biogenesis has started only recently. The process involves lipid rearrangements regarding their asymmetry on the plasma membrane promoted by membrane translocases, scramblases, and calpain, which drives the exposure of phosphatidylserine, elevation of cytosolic Ca^++^ levels, an increase in the curvature of the membrane toward the outer leaflet, and consequent cytoskeleton disassembly (Kalra et al., 2016). Cargo sorting occurs by two pathways: membrane-associated cargo is driven to the sites of budding by anchoring to the plasma membrane or affinity of lipid rafts, and cytosolic cargo has to be bound to the inner leaflet of the site, which will subsequently bud (van Niel et al., 2018).

Biogenesis of the microvesicles appears to be related to cholesterol, an abundant component of these vesicles, since pharmacological depletion of cholesterol impairs ectosome formation (Del Conde et al., 2005). RHO GTPases and RHO-associated protein kinases regulate actin dynamics and have been associated with biogenesis process (van Niel et al., 2018). Other mechanisms for microvesicle origin have been described, including microvesicles released from platelets with negative annexin-V staining, suggesting that phosphatidylserine exposure associated with translocation of amino phospholipids is not imperative (Exner et al., 2010). Furthermore, the components of the ESCRT machinery participate in the release of the microvesicles. Arrestin domain containing protein-1 (ARRDC-1) binds to the plasma membrane, displaces TSG101 from the endosomal pathway to the plasma membrane, and promotes the release of ARRDC1^+^/TSG101^+^/CD63^−^ microvesicles (Nabhan et al., 2012). Finally, microvesicle release can also be mediated by the small GTP-binding proteins ARF6 and ARF1, which act on the myosin light chain and promote the release of the vesicles (Nabhan et al., 2012; Teng and Fussenegger, 2020).

The cargo of the microvesicles is quite similar to that of the exosomes, including metalloproteins, glycoproteins, adhesion receptors, cytoskeletal components, chaperones, lipids (predominantly phosphatidylcholine, sphingomyelin, and phosphatidylethanolamine), cell-type specific proteins, RNAs, noncoding RNAs, and DNAs (van Niel et al., 2018). As microvesicles are originated by the budding of the plasma membrane, components of this cell compartment are present in large EVs. However, investigation of the composition of the microvesicles requires scrutiny, since their content may vary in enrichment depending on the parental cells and target environments. Finally, the microvesicles can be described by other terms, such as ectosomes, oncosomes, microparticles, and shedding vesicles.

## Extracellular Vesicles as Pivotal Players in Biological Processes

### Extracellular Vesicles in Invader-Host Interplay: Prominent Prototypes

EVs may contribute to biofilm formation. *Pseudomonas aeruginosa* is a Gram-negative bacterium associated with a series of opportunistic infections, especially in immunocompromised patients, cystic fibrosis patients and patients in intensive care units (Kerr and Snelling, 2009). Importantly, infection by *P. aeruginosa* has a poor prognosis because the pathogen may form a biofilm inside the host, which is intrinsically resistant to most antibiotics and is of concern due to an increase in the number of resistant strains (Tacconelli et al., 2018). EVs secreted by *P. aeruginosa* (also known as outer membrane vesicles (OMVs) contain virulence factors, such as peptides, which contribute to biofilm formation, protecting growing bacteria from the host immune response and antibiotic action (Esoda and Kuehn, 2019). Additionally, *P. aeruginosa-*released EVs downregulate the expression of MHC-related molecules in pulmonary macrophages, reducing pathogen clearance (Armstrong et al., 2020). Also, EVs released by *P. aeruginosa-*infected microglia cells diminish cell viability and the expression of the CC-chemokine ligand CCL4 by uninfected microglia cells (Jones et al., 2019). Virulence factors detected in EVs are protected from degradation in the host milieu and enable the communication and exchange of the contents within resident bacterial population or may act as competition factors with other bacteria *in situ*, favoring tissue colonization by *P. aeruginosa* (Mashburn and Whiteley, 2005). Conversely, during *P. aeruginosa*-induced pneumonia, host cells in the bronchoalveolar fluid secrete EVs that help to eliminate the infection, since uptake of these EVs by alveolar macrophages promotes M1 polarization, inflammasome activation, and neutrophil recruitment (Lee et al., 2019). Thus, these features and functions enhance the role of EVs as a promising tool for antibiotic delivery systems and natural or bioengineered vaccines for *P. aeruginosa* infection control (Schulz et al., 2018; Wang et al., 2019; Chen et al., 2020).

EVs are pivotal players in viral infections. Human immunodeficiency virus (HIV), the causative agent of acquired immunodeficiency syndrome (AIDS), affects unequally people in virtually all countries worldwide, and 38 million people have been estimated to live with this virus by the end of 2019 (Challacombe, 2020). Despite the prevention of the manifestation of AIDS, available antiretroviral therapies do not preclude chronic inflammation (Pérez et al., 2019). Fascinatingly, rather controversial roles of EVs in viral infections have been described, such as inhibition of viral replication, enhancement of viral infection, induction of viral escape from the host immune response, and cross-transfer of the components between the viruses and host cells (Kumar et al., 2020). For instance, HIV virions may take over the host exosome machinery and package the virus capsid, since retroviruses and exosomes share common proteins both in their biogenesis and target mechanisms. This sharing has been described in activated T cells, macrophages, and dendritic cells, in which released EVs participate in the transinfection process (Gould et al., 2003). In this case, EVs containing virus particles and exosomal proteins activate multiple molecules, initiate a productive infection of CD4^+^ T cells, and upregulate the expression of proinflammatory cytokines, such as interferon (IFN)-γ, tumor necrosis factor (TNF), interleukin (IL)-1β, and CCL5 (Nguyen et al., 2003; Booth et al., 2006; Wiley and Gummuluru, 2006; Izquierdo-Useros et al., 2009; Kulkarni and Prasad, 2017). Additionally, EVs can promote the transfer of the chemokine receptors CCR5 and CXCR4, which act as mediators of HIV entrance into the host cells, to the cells devoid of these receptors, fueling HIV infection (Mack et al., 2000; Rozmyslowicz et al., 2003). EVs released from HIV-infected cells reduce the ability of CD4^+^ T cells to block the entry of the viruses and promote apoptosis, favoring HIV infection (Lenassi et al., 2010; de Carvalho et al., 2014). On the other hand, infected cells can transfer certain proteins to uninfected T cells via EVs, thus preventing HIV replication in these cells *in vitro* (Khatua et al., 2009). Importantly, EVs that impair viral infection have been detected in body fluids, such as vaginal mucus, semen, and breast milk (Madison et al., 2014; Näslund et al., 2014; Smith and Daniel, 2016). The central nervous system (CNS) is a crucial site of HIV persistence and replication (Carroll-Anzinger and Al-Harthi, 2006). HIV may enter the brain via Trojan-horse macrophages (Fischer-Smith et al., 2008), infecting glial cells and stimulating microglia cell migration, proinflammatory cytokine release, neurotoxicity, blood-brain barrier impairment, and amyloid beta (Aβ)-peptide deposition (Rahimian and He, 2016; Raymond et al., 2016; András et al., 2017; Yang et al., 2018). Notably, synaptic injury, inflammation, and stress response markers were detected in cerebrospinal fluid EVs of HIV^+^ patients with cognitive impairment (Guha et al., 2019).

### Extracellular Vesicles in Neurodegenerative Diseases: Alzheimer Disease

EVs in brain physiology and neurodegeneration. In the CNS, EVs are related to the maintenance of homeostasis, mediating neuron-glial cells communication (Lachenal et al., 2011; Frühbeis et al., 2013; Drago et al., 2017; Pascua-Maestro et al., 2018). However, EVs released by neurons may contribute to pathological conditions as Alzheimer disease (AD), a neurodegenerative disorder featured by the presence of senile plaques composed mainly of Aβ-peptide depositions and neurofibrillary tangles (Song et al., 2020). EVs enriched in Aβ-peptides and neurofibrillary tangles have been detected in the brain of AD patients, and higher levels of Aβ are present in the plasma of dementia-stage AD patients compared to that in AD patients with mild cognitive impairment, suggesting either a higher pathogenicity of EVs in disease progression or a lower clearance capacity (Winston et al., 2016; Ngolab et al., 2017). Studies in mouse models of AD suggest that EVs may systemically disseminate Aβ-peptide (Perez-Gonzalez et al., 2012; Rosas-Hernandez et al., 2019). Further, microglia cells may release in EVs cargo phagocytosed toxic forms of Aβ-peptide and neurofibrillary tangle and contribute to senile plaque formation and neuronal damage (Joshi et al., 2014; Asai et al., 2015). Neuroinflammation, a hallmark of AD, is a complex process. EVs cargo released by inflammatory microglia cells contain upregulated levels of proinflammatory miRNAs and downregulated levels of anti-inflammatory miRNAs, contributing to AD pathogenesis (Gao et al., 2019). Conversely, microglia cells control the clearance of toxic peptides through the uptake of neuronal EVs containing Aβ-peptide (Yuyama et al., 2012). This imbalance in EV activity may be related to AD progression; and the molecular mechanisms regulating these processes need to be elucidated. Additionally, EVs may have therapeutic potential for AD, crossing the blood-brain barrier and acting as drug or gene delivery systems. In mouse models, mesenchymal stem cell-born and miRNA-carrying EVs were efficient in ameliorating cognitive functions, reducing the senile plaques and proinflammatory cytokine levels and promoting the upregulation of Aβ-peptide clearance factors (Ding et al., 2018; Jahangard et al., 2020).

### Heart is a Minefield by EVs

The deposition of amyloid peptides in the vascular and cardiac tissues in the elderly has been linked to inflammation and organ dysfunction, which may contribute to chronic heart failure (CHF) onset (van den Berg et al., 2019). CHF is a progressive condition affecting more than 26 million people worldwide, especially the elderly, but also a proportion of younger patients, reducing the quality of life (Savarese and Lund, 2017). Hypertension, type 2 diabetes mellitus, HIV infection, and various types of cardiomyopathies are the risk factors associated with CHF (Triposkiadis et al., 2020). The secretion of EVs containing miRNAs associated with disruption of redox signaling and hypertrophic gene expression was described *in vitro* in cardiac fibroblasts and cardiomyocytes in response to stimulation with TNF, angiotensin-II, norepinephrine, and transforming growth factor (TGF)-β, which are substances related to CHF (Bang et al., 2014; Tian et al., 2018); traits replicated in a rat model of CHF (Tian et al., 2020). Chronic activation of the renin-angiotensin system results in cardiac hypertrophy associated with high levels of angiotensin-II. Under these conditions, cardiac fibroblasts release EVs that upregulate the renin-angiotensin system in cardiomyocytes, self-maintaining their own hypertrophic ability via an unknown pathway (Lyu et al., 2015). Thus, EVs may act as a paracrine mechanism in cardiac cells contributing to CHF onset. Compared to healthy controls, plasma of CHF patients carries larger quantities of EVs, containing mitochondrial DNA (mtDNAs) and with a proinflammatory profile. Indeed, *in vitro* these EVs stimulate the release of IL-1β and IL-8, which may explain the chronic inflammation in CHF patients (Ye et al., 2017). Also, *in vitro* skeletal muscle cells shed EVs that stimulate vascular endothelial growth factor (VEGF)-independent angiogenesis in endothelial cells, opening a therapeutic opportunity to overcome the rarefaction of the capillaries in CHF patients (Nie et al., 2019). In mouse models of CHF, therapy using delivery of stem cell-born EVs into the peri-infarct zone of the myocardium reduced proinflammatory monocyte infiltration, size of the infarcted area and systemic cytokine levels, and improved cardiac functions, which may contribute to a positive outcome (Kervadec et al., 2016; Lima Correa et al., 2020), opening a new avenue for further studies.

### Hallmarks

Participation of EVs in physiological and pathological processes described as infections by prototype bacteria and viruses, chronic neurodegenerative diseases, and cardiac disorders arouses interest in the communications of various cell types and how this communication can influence the outcome of these processes. Thus, the present review will summarize the historical features of EVs and Chagas disease caused by the protozoan parasite *T. cruzi* characterized by systemic inflammatory profile in the chronic phase and by progressive cardiac disorder that may be paralleled by psychiatric and neurocognitive alterations, leading to a decrease in the quality of life of afflicted people and to frequent premature death.

## Chagas Disease: Natural History

Over one hundred 10 years ago, Carlos Chagas described the protozoan parasite *T. cruzi*, identified as the etiological agent of an illness, named Chagas disease (CD), the insect vector (a triatomine bug) and the complete life cycle of the parasite (Rassi et al., 2010). In endemic areas in Latin America, detection of *T. cruzi* DNA in mummies refers to the pre-Columbian era (Reinhard and Araújo, 2016). The parasite life cycle is complex and is under revision. The kissing bug triatomine vector is contaminated after blood suckling. The *T. cruzi* epimastigote (Epi) forms replicate in the midgut of the insect vector and migrate to the rectum region, differentiating into metacyclic trypomastigotes (mTrypo); eventually, the infective forms are released in triatomine excretion. mTrypo forms enter the host skin through a bite wound and ocular and oral mucosa. Inside the host cells, mTrypo differentiate into amastigotes (Ama), which are the multiplicative forms in mammalian hosts. After several cycles of proliferation, Ama differentiate into trypomastigotes (Trypo); after cell rupture, Trypo can reach the extracellular milieu to infect the surrounding cells and reach the bloodstream. Bloodstream trypomastigotes (bTrypo) disseminate the infection in all mammalian host tissues and/or can be ingested with the blood during insect feeding, completing the replicative cycle (De Souza and Barrias, 2020).

Most of clinical manifestations of CD occur during the productive time of afflicted subjects, reducing family income and the quality of life, increasing mortality, and incapacitation rates (Coura and Viñas, 2010). Nevertheless, after effective integrated international actions to control the main domiciliated vector *Triatoma infestans*, the number of acute cases dropped in Brazil, Argentina, and other countries (Coura and Borges-Pereira, 2012). Thus, other means of infection gained epidemiological importance, such as congenital and oral transmission, organ transplantation and blood transfusion (Rassi et al., 2010). There are new challenges to be faced to control CD: in endemic areas are approximately 70 million people at risk of infection, 14,000 deaths per year and 14,000 congenital cases per year; and human migration worldwide increased rates of seropositive persons for *T. cruzi* infection in North America, Oceania, and European countries (The Lancet, 2019; Colombo et al., 2020). Surveillance and educational actions are required to increase awareness among citizens at risk of infection and public health decision makers to define the conditions to diagnose infected people, to properly and timely treat them with available trypanocidal drugs, and to offer integrative care (Lannes-Vieira et al., 2010; Carolina et al., 2019). In 2019, the World Health Organization has implemented the World Chagas Day, to increase global awareness of the cause of the disease and to stimulate the investments to fight the disease (Ferreira and Andricopulo, 2020).

Acute phase of *T. cruzi* infection is characterized by patent parasitemia and mostly unnoticed or mild unspecific symptoms and clinical signs. In rare cases, such as immunocompromised patients or newborns, this phase may lead to myocarditis and meningoencephalitis. In acute phase, the treatment is mostly missed; however, if used, available drugs (benznidazole and nifurtimox) can lead to cure (60–80%) or may prevent chronic determinate forms of the disease (Rassi et al., 2010). Decades after the infection, most of persons seropositive for *T.* cruzi (60–70%) are asymptomatic and show no clinical signs, corresponding to the indeterminate form (IND) of CD. However, 30–40% of infected people progress to chronic determinative forms: cardiac (CARD), digestive (DIG) and cardio-digestive (CARDDIG) (Rassi et al., 2012; Dias et al., 2016b; Nunes et al., 2018; Simões et al., 2018). Mental disorders and neurocognitive abnormalities (anxiety, depression, and memory loss) may parallel other clinical forms of chronic CD (Silva et al., 2020). These clinical forms may result from the host-parasite interplay and growing evidence indicate that the *T. cruzi* genotypes, which are classified into seven discrete typing units (DTUs) considering a set of genetic, molecular, and immunological biomarkers (Zingales et al., 2009), may also contribute to the clinical outcome; however, there is no consensus on this issue (Zingales, 2018).

## Exoantigens of *T. cruzi*: A Brief History

In recent decades, attempts to define the pathogenesis of CD have unveiled features of both the host (immunological unbalance and intrinsic tissue factors) and parasite (genetic variability and parasite load) that may influence the onset of the disease and severity of the clinical outcome. Extensive efforts have been aimed to understand the dynamics of the host-parasite interactions that lead to immune response and parasite control but not to elimination, resulting in parasite persistence in the tissues during the chronic phase of the infection, which may contribute to the clinical outcome. Initial studies demonstrated that *in vitro* released *T. cruzi* exoantigens, primarily identified to be composed of carbohydrates and proteins, present strong antigenicity *in vivo* (Tarrant et al., 1965). Later, several groups described the presence of *T. cruzi* soluble antigens in experimental infection *in vitro* and in the sera and urine of acute and chronically infected patients. These findings led to a hypothesis that parasite exoantigens may be a mechanism of evasion of the host immune response. Furthermore, several studies considered the applicability of these exoantigens for CD diagnosis (Araujo et al., 1981; Affranchino et al., 1989; Ouaissi et al., 1990; Corral et al., 1996; Gruppi et al., 1997).

**FIGURE 1:**
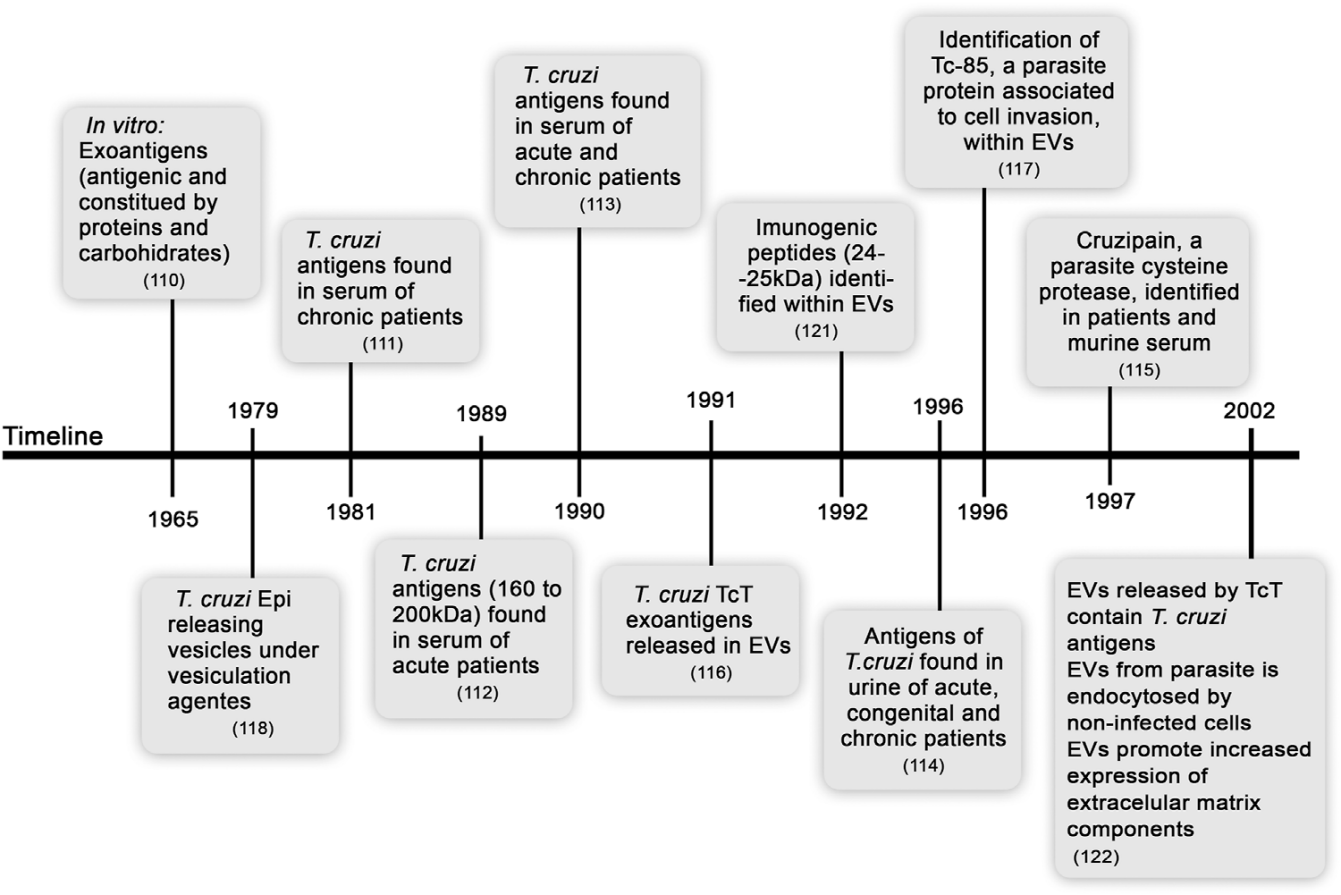
Timeline from the discovery of soluble exoantigens to description of the release of extracellular vesicles by *Trypanosoma cruzi.*

In a pioneering study, the release of *T. cruzi* exoantigens associated with membrane vesicles was shown to be a continuous and spontaneous process in cell-cultured trypomastigotes (TcT) (Gonçalves et al., 1991). The release of EVs is a process preserved in the TcT of various tested strains (CA1, Y, YuYu, and RA) and was dependent on the temperature and time of culture. EVs of all these *T. cruzi* strains contain various amounts of Tc-85 antigen, which is a molecule associated with cell invasion (Abuin et al., 1996). Years before these findings were reported, the ability of *T. cruzi* Epi to release vesicles was observed, showing the presence of glycoprotein-enriched EVs after stimulation by vesiculation agents (such as acetate, citrate, and formaldehyde) and at acidic pH (da Silveira et al., 1979). Furthermore, phosphatidylcholine and phosphatidylethanolamine were described as the main lipids of Epi EVs (Franco da Silveira and Colli, 1981). Later, the release of lipids enriched in phosphatidylcholine, lysophosphatidylcholine, and free fatty acids by *T. cruzi* TcT was shown (Agusti et al., 2000), supporting complex composition of parasite-excreted EVs. A highly immunogenic 24–25 kDa TcT peptide was detected within EVs budding from the outer membrane or derived from the flagellar pocket, showing that EVs released by various parasite structures may activate the host immune system (Ouaissi et al., 1992). These findings were corroborated by a study, which showed that antigen-containing TcT EVs are adsorbed to the cell membrane and endocytosed by uninfected cells; these cells are recognized by the sera of rabbits chronically infected with *T. cruzi*. Moreover, interactions of EVs with mammalian cells increase the expression of extracellular matrix (ECM) components, such as fibronectin, laminin, and type-I collagen (Pinho et al., 2002). These initial studies on *T. cruzi* exoantigens are summarized in Figure 1. Biological consequences of host cell-parasite EV interactions are not fully understood; however, these interactions may indicate that EVs containing released antigens contribute to long-lasting parasite persistence, perpetuate inflammation, modulate cell functions, and contribute to tissue remodeling and disease outcome.

Due to the impact of CD, considerable attention has been attracted to mammalian host-parasite interplay; however, some studies have focused on other biological roles of EVs in *T. cruzi* biology. EV function in parasite-parasite communications was observed in nutritionally stressed Epi. These EVs are rich in small RNAs and are incorporated by other Epi, stimulating parasite differentiation in a dose-dependent manner (Garcia-Silva et al., 2014a; Fernandez-Calero et al., 2015). These EVs were also capable of modulating the communications of an invertebrate host and the parasite, delaying parasite migration to the rectum of the triatomine bug *Rhodnius prolixus* (Paranaiba et al., 2019).

At present, there are no mechanistic studies focusing on biogenesis of *T. cruzi* EVs. The presence of secreted material in two different vesicle fractions, one of MVB origin and another originating from the plasma membrane budding, and a soluble fraction not associated with vesicles was described in Epi and mTrypo (Dm28c clone) (Bayer-Santos et al., 2013). Proteomic analysis revealed the differences in the protein contents between both EV fractions from various parasite forms. These findings support the idea of different mechanisms of secretion in this trypanosomatid. The process of EV release by *T. cruzi* appears to be common in most parasite forms and strains and has been observed in Epi, mTrypo, bTrypo, TcT, and extracellular amastigote-like forms; nonetheless, the absence of the studies of EVs in intracellular amastigotes is justified because their location is prone to contamination by the host cell material. The strains and forms used in these studies are summarized in Table 1.

**TABLE 1:** Extracellular vesicles shed by various strains and forms of *T. cruzi.*

Form		Strain	References
Epi		Y	da Silveira et al. (1979)
		Dm28 clone	Bayer-Santos et al. (2013); Garcia-Silva et al. (2014a); Garcia-Silva et al. (2014b)
		Pan4	Florentino et al. (2018); Retana Moreira et al. (2019)
Extra-Ama		G	Florentino et al. (2018)
		CL	Florentino et al. (2018)
		Pan4	Díaz Lozano et al. (2017)
Trypo	mTrypo	Dm28 clone	Bayer-Santos et al. (2013)
		Pan4	De Pablos et al. (2016); Díaz Lozano et al. (2017)
		CL-Brener	De Pablos et al. (2016)
		G	Clemente et al. (2016)
		CL	Clemente et al. (2016)
	TcT	Yuyu	Gonçalves et al. (1991); Nogueira et al. (2015); Ribeiro et al. (2018)
		CA-1	Gonçalves et al. (1991)
		RA	(Gonçalves et al., 1991; Caeiro et al., 2018)
		Y	Gonçalves et al. (1991); Ouaissi et al. (1992); Pinho et al. (2002); Trocoli-Torrecilhas et al. (2009); Neves et al. (2014); Nogueira et al. (2015); Caeiro et al. (2018); Lovo-Martins et al. (2018); Ribeiro et al. (2018)
		CL-Brener	Neves et al. (2014); Caeiro et al. (2018)
		Colombian	Nogueira et al. (2015)
		CL-14	Nogueira et al. (2015)
		Pan4	Díaz Lozano et al. (2017); Retana Moreira et al. (2019); Retana Moreira et al. (2021)
		Sylvio	Caeiro et al. (2018)
		173	Caeiro et al. (2018)
	bTrypo	RA	Caeiro et al. (2018)

Epi, epimastigotes; Extra- Ama, extracellular amastigote-like; Trypo, trypomastigotes; mTrypo, metacyclic trypomastigotes; TcT, tissue-cultured derived trypomastigotes; bTrypo, bloodstream trypomastigotes.

## Extracellular Vesicles Modulating the Immune Response in *Trypanosoma cruzi* Infection

CD is a complex illness that may lead to severe outcomes related to cardiac and gastrointestinal clinical manifestations that may also be associated with psychiatric illness and cognitive deficit. In the acute phase of infection, control of parasite replication is critical for host survival, allowing the transition into the chronic phase. However, parasite control is partial, and intermittent parasitemia and low-grade parasitism are present independently of the clinical outcome. Variable intensity of tissue inflammation, intrinsic tissue alterations (e.g., mitochondrial damage and oxidative stress), and remodeling are associated with this phenomenon and may contribute to the onset and progression of defined clinical forms of chronic CD. Moreover, the progression of the disease appears to be associated with an unbalanced systemic immune response and response in the tissues target of parasite persistence. Moreover, the progression of the disease appears to be associated with an unbalanced immune response, systemically and in tissues targets of parasite persistence. However, cellular and molecular mechanisms of pathogenesis of CD are incompletely understood (Rassi et al., 2010; Dutra et al., 2014; Dias et al., 2016b; Chevillard et al., 2018).

The release of EVs is a process conserved in evolution. The recognition of EVs by immune and nonimmune cells plays a role in the regulation of the immune response through juxtracrine and/or paracrine signaling, since EV cargo can be transferred to acceptor cells (Wen et al., 2017). For instance, B cells release EVs containing MHC-II, costimulatory and cell adhesion molecules, which can directly or indirectly stimulate T cells. These EVs can be captured by follicular dendritic cells, which do not express MHC-II, to incorporate this receptor into their cell membrane (Robbins et al., 2016). Interestingly, mRNA cargo in EVs shed by mouse mast cells delivered to recipient human mast cells is translated into proteins, opening an opportunity to regulate the immune response by modifying gene expression and protein production in the target cells (Valadi et al., 2007). In murine model of acute CD (Navarro et al., 2015) and in patients with chronic chagasic cardiomyopathy (Laugier et al., 2020) several miRNA and mRNA are differentially expressed in the heart tissue, which could influence the clinical outcome of CD. The integrative view of these altered mRNA and miRNA in *T. cruzi*-infected mice and patients revealed TNF, IFN-γ, and NF-kB as upstream regulators of pathways/processes associated with fibrosis, arrythmia, mitochondrial damage, and inflammation (Ferreira et al., 2017; Laugier et al., 2020). The main actors triggering these processes are still not fully understood and, therefore, it is reasonable to consider that EVs may be taking part in these changes.

As discussed above, various *T. cruzi* strains and life stages release EVs, which may interfere with parasite-parasite and parasite-host cell interactions via various pathways. Despite simultaneous occurrence of all these processes, we separately discuss the effects promoted by 1) EVs released by the parasite; 2) EVs obtained from the blood of CD patients; 3) EVs shed by interactions of the parasite and host cells; and 4) EVs released by infected host cells.

### EVs Released by the Parasite

*T. cruzi* releases variable amounts of EVs with distinct protein and carbohydrate contents depending on the strain and evolutive form of the parasite (Bayer-Santos et al., 2013; Nogueira et al., 2015; Ribeiro et al., 2018; Retana Moreira et al., 2021). TcT of the YuYu strain releases a high amount of EVs enriched in proteins related to virulence, and EVs shed by the parasites of the Y strain contain virulence-associated proteins and are enriched in carbohydrates (Nogueira et al., 2015; Ribeiro et al., 2018). In the case of the Dm28 clone, EVs released by Epi are enriched in nucleic acid-binding proteins compared to EVs released by mTrypo (Bayer-Santos et al., 2013). Interestingly, peritoneal macrophages stimulated with EVs derived from the YuYu strain produce nitric oxide (NO), TNF and IL-6, and EVs shed by the parasites of the Colombian strain do not induce significant levels of cytokines and NO; however, these strains belong to the same genetic group (DTU Tc I) (Nogueira et al., 2015). Even extracellular Ama-like forms can release EVs containing proteins, such as ssp-4, which can be recognized by the target cells and mediate parasite entry (Florentino et al., 2018). Diverse cargo within EVs is protected from extracellular degradation and the action of the immune system until it reaches the target cells and may constitute a conserved strategy for remote delivery of effector molecules that can favor parasite survival and infection (Coakley et al., 2015).

**FIGURE 2:**
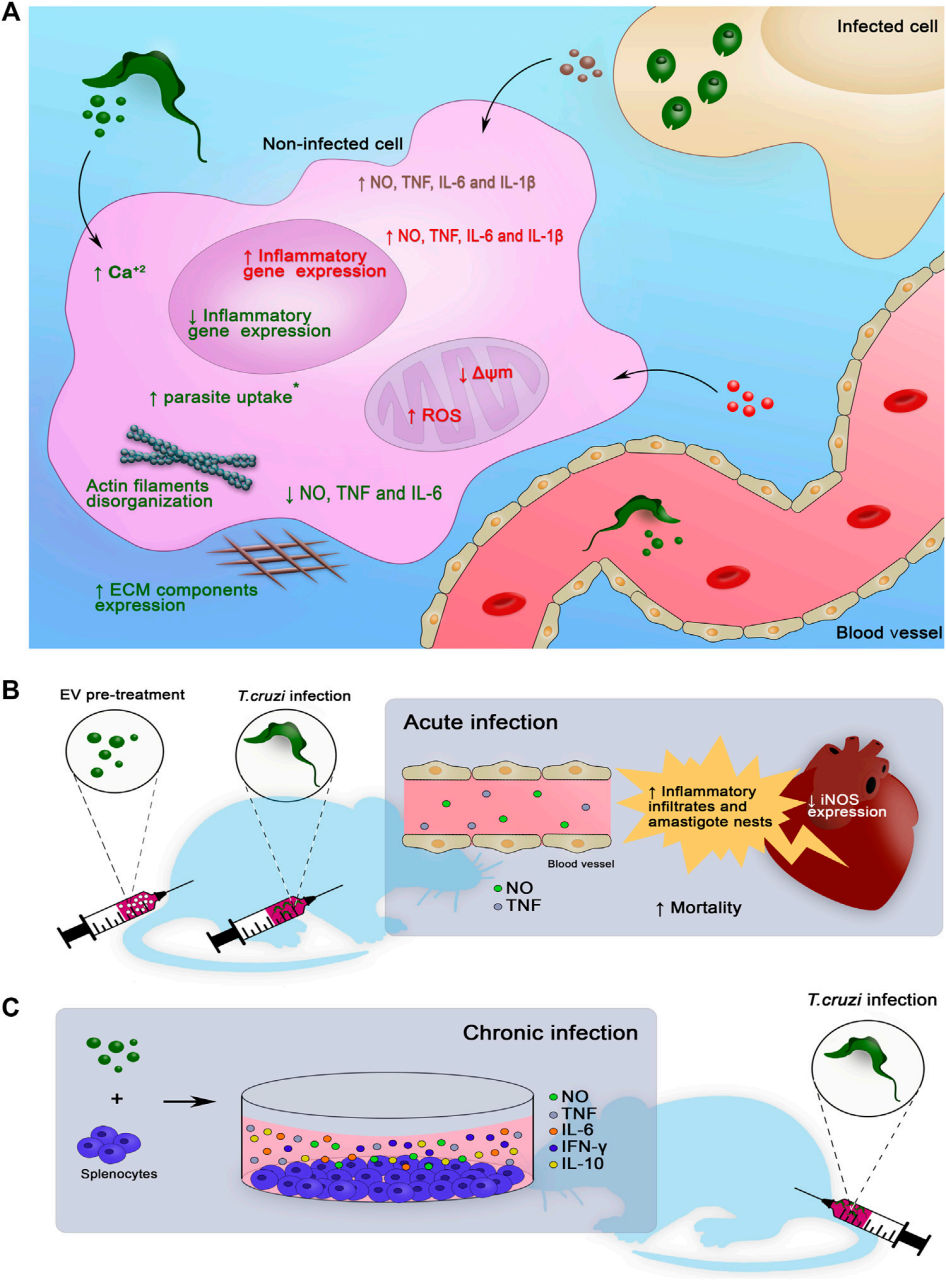
Main regulatory activities of EVs from various sources in a scenario of *Trypanosoma cruzi* infection analyzed *in vitro* **(A)**, *in vivo* **(B)** and *ex vivo* **(C)**. **(A)** EVs released by the parasite have been shown to promote increased transient intracellular calcium levels (Retana Moreira et al., 2019) and actin filament disorganization (Retana Moreira et al., 2019), modulate the expression of extracellular matrix components (Pinho et al., 2002; Retana Moreira et al., 2019), reduce the gene expression of proinflammatory cytokines (Cronemberger-Andrade et al., 2020) and reduce the release of NO, TNF, and IL-6 when in contact with uninfected cells (Nogueira et al., 2015; Lovo-Martins et al., 2018). The treatment with these EVs before infection with *T. cruzi* promotes an increase in parasite uptake in a target cell type-dependent manner (*) (Lovo-Martins et al., 2018). EVs released either by immune or nonimmune infected cells promote an increase in NO, TNF, IL-6, and IL-1β levels in uninfected cells (Choudhuri and Garg, 2020a). EVs from the blood sources obtained from CD patients promote an increase in NO and IL-1β release, increase mitochondrial ROS levels, and decrease mitochondrial membrane potential and inflammatory gene expression in EV-treated macrophages (Chowdhury et al., 2017). The treatment of macrophages with EVs obtained from the blood of mice with chronic infection increases TNF, IL-6, and IL-1β release (Choudhuri and Garg, 2020a). **(B)** BALB/c mice pretreated with EVs from *T. cruzi* and challenged by infection have reduced NO and TNF levels in the serum in the acute phase (Trocoli-Torrecilhas et al., 2009; Lovo-Martins et al., 2018), reduced iNOS expression (Trocoli-Torrecilhas et al., 2009), increased number of inflammatory infiltrates and amastigote nests in heart tissue (Trocoli-Torrecilhas et al., 2009; Lovo-Martins et al., 2018), and increased mortality (Trocoli-Torrecilhas et al., 2009). **(C)** Splenocytes obtained from chronically *T. cruzi*-infected C57Bl/6 mice treated with parasite EVs showed increased NO, TNF, IL-6, IFN-γ, and IL-10 release (Nogueira et al., 2015), contributing to the idea that EVs participate in CD pathogenesis.

Previous contact with EVs released by *T. cruzi* parasites induces certain changes in the target cells allowing parasite infection, such as a transient increase in the intracellular calcium, actin filament disorganization, cell cycle arrest, cell permeabilization, and expression of ECM genes and proteins (Garcia-Silva et al., 2014b; Retana Moreira et al., 2019). Treatment of bone marrow-derived macrophages with EVs shed by the TcT parasites induces prostaglandin E_2_ (PGE_2_) and lipid bodies formation. After *T. cruzi* infection, these EV-treated macrophages decrease the production of PGE_2,_ TNF, and IL-6. Furthermore, parasite uptake and infective TcT release were increased in macrophages pretreated with EVs (Lovo-Martins et al., 2018). Additionally, the interaction of human macrophages with *T. cruzi* EVs decreases the gene expression of proinflammatory cytokines (IL-1β and IL-6) and Toll-like receptor (TLR) 2 (Cronemberger-Andrade et al., 2020). The production of proinflammatory cytokines (TNF, IL-6, IL-12, and IL-1β) and NO by infected cells is crucial to infection resistance (Aliberti et al., 1996; Hölscher et al., 1998; Shoda et al., 2001) and can be triggered via TLR2 activation by parasite-derived molecules, such as glycosylphosphatidylinositol (GPI) anchors and/or glycoinositol phospholipids (GIPLs) (Campos et al., 2001; Camargo et al., 1997; Almeida et al., 2000). Increased levels of PGE_2_ have been linked to susceptibility to *T. cruzi* infection and stimulation of parasite replication (Cardoni and Antúnez, 2004; D’Avila et al., 2011). Therefore, EVs shed by *T. cruzi* in the extracellular milieu appear to exert a modulatory effect on the host cells prior to infection, favoring parasite infection and persistence (Figure 2A). However, it is not clear whether this effect depends on the host cell type. Interestingly, peritoneal macrophages produce IL-12, NO, TNF, and IL-10 after TcT-EV stimulus. A second stimulus increases the production of IL-10, IL-12, and NO to a lesser extent; however, TNF production was hampered. This response with a regulatory profile can induce a Th2 response of T cells, inhibit the activation of macrophages, and consequently enhance parasitism (Trocoli-Torrecilhas et al., 2009). This idea was corroborated by findings in EV-pretreated peritoneal macrophages to decrease the frequencies of CD11b^+^/MHC-I^+^/MHC-II^+^ cells and NO levels and to increase internalization of the parasites (Lovo-Martins et al., 2018). This study also shows that reduced plasma levels of TNF and NO were detected in acutely infected mice pretreated with EVs (Lovo-Martins et al., 2018), reinforcing the importance of the results obtained in the *in vitro* study (Figure 2B).

EV endocytosis by the target cells has been postulated (Pinho et al., 2002; Bayer-Santos et al., 2013), and one of the interaction pathways proposed occurs via glycoconjugates and/or enzymatic activity (Retana Moreira et al., 2019). Indeed, the presence of acidic and alkaline phosphatases in EVs has been demonstrated to interfere with *T. cruzi* infection of the host cells (Neves et al., 2014). Although different pathways may be implicated in *T. cruzi* invasion promoted by EVs, experimental evidence supports that parasite invasion via TLR2 signaling may be enhanced (Nogueira et al., 2015; Cronemberger-Andrade et al., 2020), most likely in a process mediated by GPI anchors and/or GIPLs within EVs (Campos et al., 2001; Bayer-Santos et al., 2013). Other EV cargo may contribute to the mechanism of this process: tsRNA (small RNA derived from tRNA) can regulate the genes involved in the immune response, ECM components, and cytoskeleton pathways (Garcia-Silva et al., 2014b) and the presence of other proteins, such as TcPIWI, may form a gene silencing complex in the target cells (Bayer-Santos et al., 2014); the mechanisms of these processes need to be further explored in EVs released by *T. cruzi*. Notably, virulence factors within EVs, such as TcSMP (Martins et al., 2015), mucin-associated surface proteins (MASPs), and trans-sialidases (TSs), can favor the infection by *T. cruzi* (Bayer-Santos et al., 2013). Other factors, such as the virulence factor Tc-TASVC present in bTrypo EVs, have been considered vaccine targets. Indeed, the Tc-TASVC DNA prime-protein boost scheme improves the survival of vaccinated mice after the challenge (Caeiro et al., 2018). Other factors identified in mTrypo EV cargo, such as gp90 specifically detected in distinct *T. cruzi* strains, downregulate host cell infection. Other factors identified in mTrypo EV cargo, such as gp90 differentially detected in distinct *T. cruzi* strains, downregulate host cell infection (Clemente et al., 2016). Complex regulatory mechanism of EVs directly released by *T. cruzi* parasites appears to be dependent on the cell type, parasite form, and strain. Preincubation with EVs from the Y strain increases the percentage of infected macrophages but does not increase the percentage of infected epithelial cells (Lovo-Martins et al., 2018; Ribeiro et al., 2018). On the other hand, EVs derived from the YuYu and Pan4 parasite strains enhance the percentage of infected epithelial cells and intracellular parasitism (Ribeiro et al., 2018; Retana Moreira et al., 2019). Thus, additional studies are required to identify molecular mechanisms governing the outcome of the biological interplay between *T. cruzi*-derived EVs and the diversity of hosts cells present in the tissues and organs.

As expected, additional complexity was detected when the effects of *T. cruzi*-derived EVs were studied in mammalian hosts. Exposure of mice to parasite EVs prior to acute infection increases the number of inflammatory infiltrates and amastigote nests in the heart tissue and augments the death rate in susceptible BALB/c mice, which was not observed in resistant C57BL/6 mice (Lovo-Martins et al., 2018; Trocoli-Torrecilhas et al., 2009) (Figure 2B). In both cases, downregulation of the immune response was observed in EV-treated mice, which have reduced serum levels of NO and TNF and decreased inducible nitric oxide synthase (iNOS) expression in the heart tissue, hampering parasite control (Nogueira et al., 2015; Lovo-Martins et al., 2018). Considering that *T. cruzi* EVs are released continuously (Gonçalves et al., 1991), it is possible to speculate that EV cargo may be different in the acute and chronic phases of the infection and in patients with diverse clinical forms of CD; thus, EV cargo plays distinct regulatory roles in the immune response (Nogueira et al., 2015; Díaz Lozano et al., 2017; Lovo-Martins et al., 2018). This idea may be particularly relevant considering that proinflammatory (IFN-γ, TNF, and IL-6) and regulatory (IL-10) cytokines (Hölscher et al., 2000; Roffê et al., 2012) may play opposite roles in immunopathological mechanisms crucial for CD progression and severity (Figures 2B,C).

The formation of immune complexes containing immunoglobulins (Ig) associated with parasite-released antigens, including those within EVs, was observed in chronic CD patients (Díaz Lozano et al., 2017). The clearance of immune complexes, which can deposit and promote tissue injury, involves the components of the complement system and contributes to inflammation, increased phagocytosis, and lysis of the pathogen (Lidani et al., 2017). Investigation of the participation of *T. cruzi* EVs in this intricate scenario has started. EV subpopulations from various *T. cruzi* strains and life stages contain MASPs (both in the immature and mature forms) that are recognized by Igs of the G subclass (IgG) of chronic CD patients, which inhibit complement-mediated parasite lysis, especially in the serum from CARD patients (De Pablos et al., 2016; Díaz Lozano et al., 2017), confirming that this phenomenon may be a part of a parasite evasion strategy. Interestingly, vaccine candidate containing MASP was tested in an experimental model of acute CD and induces a balanced proinflammatory/regulatory (IFN-γ, IL-12, IL-17, and IL-10) immune response, promoting host survival, and reducing parasite load in the heart, liver, and spleen (Serna et al., 2014) but not clearing the infection. In this context, in an experimental model of chronic CD, a fast early humoral response with increased levels of IgM against MASP peptides from *T. cruzi* EV cargo was observed; however, this response failed to promote a sufficient transition to IgG. Thus, EVs continuously released by *T. cruzi* act in innate and acquired immune responses, inhibiting the complement system and evading the humoral immune response (De Pablos et al., 2016). Overall, these data reinforce the fact that parasite-shed EVs can modulate the host immune response, which gains complexity considering a possible contact of mammalian hosts with various parasite stages and strains (Table 1).

### EVs Obtained From the Blood of CD Patients

Recent search for biomarkers of clinical stages, disease severity, and prognosis led to the studies on phenotypical and functional characterization of EVs in the peripheral blood of CD patients. Initial work demonstrated that EVs collected from the blood of chronic patients primarily originate from macrophages/monocytes and lymphocytes (Chowdhury et al., 2017). Furthermore, EVs in the blood of clinically asymptomatic and symptomatic CARD patients increase mitochondrial ROS production and NO and IL-1β release, and reduce the mitochondrial membrane potential in the human THP-1 macrophage cell line (Figure 2A). However, distinct gene profiles are expressed by these cells. EVs from symptomatic CARD patients increase the inflammatory pattern, and EVs from asymptomatic CARD patients induce a mild-to-moderate inflammatory profile (Chowdhury et al., 2017). It has been shown that EVs work as proinflammatory signal amplifiers in the neighboring cells, fueling oxidative/nitrosative stress observed in patients starting from the early stages of CD (Carrasco Guerra et al., 1987). Circulating EVs of chronic CD patients are similar in size but have lower concentrations than those of healthy individuals. Additionally, stratification of CD patients revealed that a reduction in EV concentration is associated with the severity of cardiac clinical parameters (Madeira et al., 2021). These differences may be related to differential release or retention of EVs in the tissues during disease progression, which may be a marker or contributor to CD pathogenesis. Therefore, circulating EVs apparently reflect the levels of oxidative/nitrosative stress and inflammatory state in CD patients. Furthermore, these EVs may be used as tools to grade disease severity and to follow the efficacy of trypanocidal or cardioprotective therapies and vaccines; these issues require additional exploration.

### EVs Shed by the Interaction of the Parasite and Host Cells

Interaction of the parasites with the host cells for a short period of time stimulates EV release by the host cells in a Ca^+2^-dependent manner (Cestari et al., 2012). Various strains (RA, Y, G, CL-Brenner, and Sylvio X10/6) and stages of the parasite stimulate the shedding of EVs from immune cells (Tables 2–4). These EVs attach to the parasite surface, forming a complex with C3 convertase and inhibiting the lysis mediated by the complement system (Cestari et al., 2012; Ramirez et al., 2017). Intriguingly, the mechanism of immune system evasion is strain-dependent, since EVs derived from the host cells after contact with the parasites of the Y strain (DTU- TcII) do not protect the parasites of the G strain (DTU-TcI) from the complement-mediated lysis (Wyllie and Ramirez, 2017). These EVs of the host cell origin contain host proteins and lipids, as expected, and parasite molecules, suggesting the fusion of EVs released from both organisms (Ramirez et al., 2017). This fact requires careful analysis since 1) EVs obtained from the supernatant may be a mixture of the vesicles of different origins and do not necessarily indicate their fusion; 2) the parasites release EVs continuously (Gonçalves et al., 1991); 3) no specific marker was used to differentiate EVs released by the parasite and EVs released by the host cells participating in the interaction; 4) cell linker used in the study binds to EV lipids and may influence their functionality or may be transferred to acceptor cells or other EVs, confounding the results (Carpintero-Fernández et al., 2017); and 5) only a few reports are available on the subject, and firm conclusions cannot be drawn. Nevertheless, EVs shed after parasite-host cell interactions favor the infection of unstimulated cells and increase the number of intracellular parasites (Cestari et al., 2012). This process is also dependent on the parasite form, since EVs obtained from the contact of *T. cruzi* Epi with the host cells do not contribute to the infection of other cells (Cestari et al., 2012; Wyllie and Ramirez, 2017). Finally, TGF-β-bearing EVs released from monocytes and lymphocytes promote rapid cell invasion by *T. cruzi*, allowing escape of the parasites from the complement attack (Cestari et al., 2012). Interestingly, previous studies confirmed that *T. cruzi* takes up host TGF-β to promote cell infection and control its own intracellular cycle (Waghabi et al., 2005). Recently, the TGF-β signaling pathway has been shown to be crucial to the formation of fibrosis in chronic Chagas cardiomyopathy (Ferreira et al., 2019). Thus, after a contact with the parasite, host cell-shed EVs may contribute to *T. cruzi* evasion of the innate immune response, promote parasite infection/differentiation, and potentially function as a system for the delivery of mediators, such as the cytokine TGF-β, favoring the deposition of the ECM content associated with fibrosis and contributing to the pathogenesis of Chagas’ heart disease.

**TABLE 2:** EVs originating from the interaction between the host cells and parasites.

Form	Strain	Interaction cells	Treated cells	References
Epi, mTrypo, TcT	Sylvio X10/6, RA, CL-Brener, G, Y	THP-1, PBMC, Jurkat	Vero	Cestari et al. (2012)
	Y, CL-Brener, Sylvio X10/6	THP-1	Vero	Ramirez et al. (2017)
mTrypo	Y, G	THP-1	Vero	Wyllie and Ramirez, (2017)

Epi, epimastigotes; mTrypo, metacyclic trypomastigotes; TcT, tissue-cultured trypomastigotes.

**TABLE 3:** EVs originating from infected cells.

Form	Strain	Interaction cells	Treated cells	References
TcT	Sylvio X10/4	PBMC	THP-1	Chowdhury et al. (2017)
	Y	THP-1	THP-1, CHO	Cronemberger-Andrade et al. (2020)
Trypo	Sylvio X10/4	Raw 264.7, C2C12	Raw 264.7, BM-derived Mφ	Choudhuri and Garg, (2020a)

Trypo, axenic trypomastigotes; TcT, tissue-cultured trypomastigotes; BM-derived MΦ, bone marrow-derived macrophages.

**TABLE 4:** EVs detected in blood.

Origin	Strain	Clinical form	Treated cells	References
Mouse	Sylvio X10/4	Chronic	Raw 264.7	Choudhuri and Garg, (2020a)
		Chronic	Raw 264.7	Chowdhury et al. (2017)
Human	—	NI, SymCARD, AsymCARD	THP-1	Chowdhury et al. (2017)
		NI, IND, CARD	THP-1	Madeira et al. (2021)
		NI, IND, CARD	—	Ramirez et al. (2017)

NI, uninfected; IND, indeterminate form; CARD, cardiac form; SymCARD, symptomatic cardiac form; AsymCARD, asymptomatic cardiac form.

The shedding of EVs from the cells after a contact with the parasites was also detected in the circulating blood of acutely *T. cruzi*-infected mice (Cestari et al., 2012). Furthermore, the treatment of mice with EVs derived from the host cell-parasite contact prior to the infection either delays or decreases parasitemia in a manner dependent on the parasite form used for the interaction (Cestari et al., 2012; Ramirez et al., 2017). Importantly, the sera of CARD patients recognize a higher number of cargo proteins contained in TcT EVs-host cells than the number of cargo proteins contained in mTrypo EVs-host cells (Ramirez et al., 2017). This differential ability to recognize the parasite antigens contained in EVs may reflect the physiopathological features of CD, which is a subject for further exploration.

### EVs Released by Infected Host Cells

EVs shed from *T. cruzi*-infected cells regulate the immune response. In uninfected cells, these EVs induce the expression of proinflammatory genes and increase the levels of TNF, IL-1β, IL-6, and NO, confirming that EVs originating from infected cells may modulate the activation of the neighboring cells (Cronemberger-Andrade et al., 2020; Chowdhury et al., 2017) (Table 3). A proinflammatory stimulation of uninfected cells can be induced both by EVs released from infected immune (macrophages) and nonimmune (muscle) cells or by EVs obtained from the blood of chronically infected mice (Choudhuri and Garg, 2020a) (Figure 2A). Evidence points out that the mechanism of action of these EVs involves TLR2, PARP1-cGAS-NF-κB (poly ADP-ribose polymerase 1- cyclic GMP-AMP synthase-NF-κB), and proteins and genetic material of the parasite (kDNA and 18S rDNA) in the EV cargo (Choudhuri and Garg, 2020a; Cronemberger-Andrade et al., 2020). Thus, EVs from *T. cruzi*-infected cells may contribute to the maintenance of proinflammatory response in the injured tissues during the course of the infection and progression of CD. The main effects of EVs on the immune response are summarized in Figure 2.

These types of EVs are formed when the host is exposed to *T. cruzi* infection (parasite-derived EVs, infected cell-derived EVs, and EVs derived from the cell-parasite interactions), as discussed separately, and simultaneous studies of these EVs yielded interesting results. For instance, when EVs were collected from the supernatant of the cells 5 days post infection, the contents include proteins of distinct *T. cruzi* stages (Ama and Trypo) and host cells (Bautista-López et al., 2017). This result was expected since sufficient incubation time allows the parasites to interact with the host cells, infect them, proliferate intracellularly, differentiate, disrupt the cells, reach the extracellular medium, and restart the cycle; therefore, EVs of distinct origins were analyzed. Furthermore, corroborating other data that EV contents are immunogenic and antigenic, the sera from clinically asymptomatic patients are less reactive against proteins of these types of EVs and the sera from CARD patients with electrocardiograph abnormalities or ventricular arrythmia are very reactive to EVs (Bautista-López et al., 2017). Thus, the study of EVs from *T.* cruzi may contribute to the understanding of CD pathogenesis and may pave the way to identification of possible disease progression and therapeutic efficacy biomarkers and vaccine candidates.

## Concluding Remarks and Limitations

**FIGURE 3:**
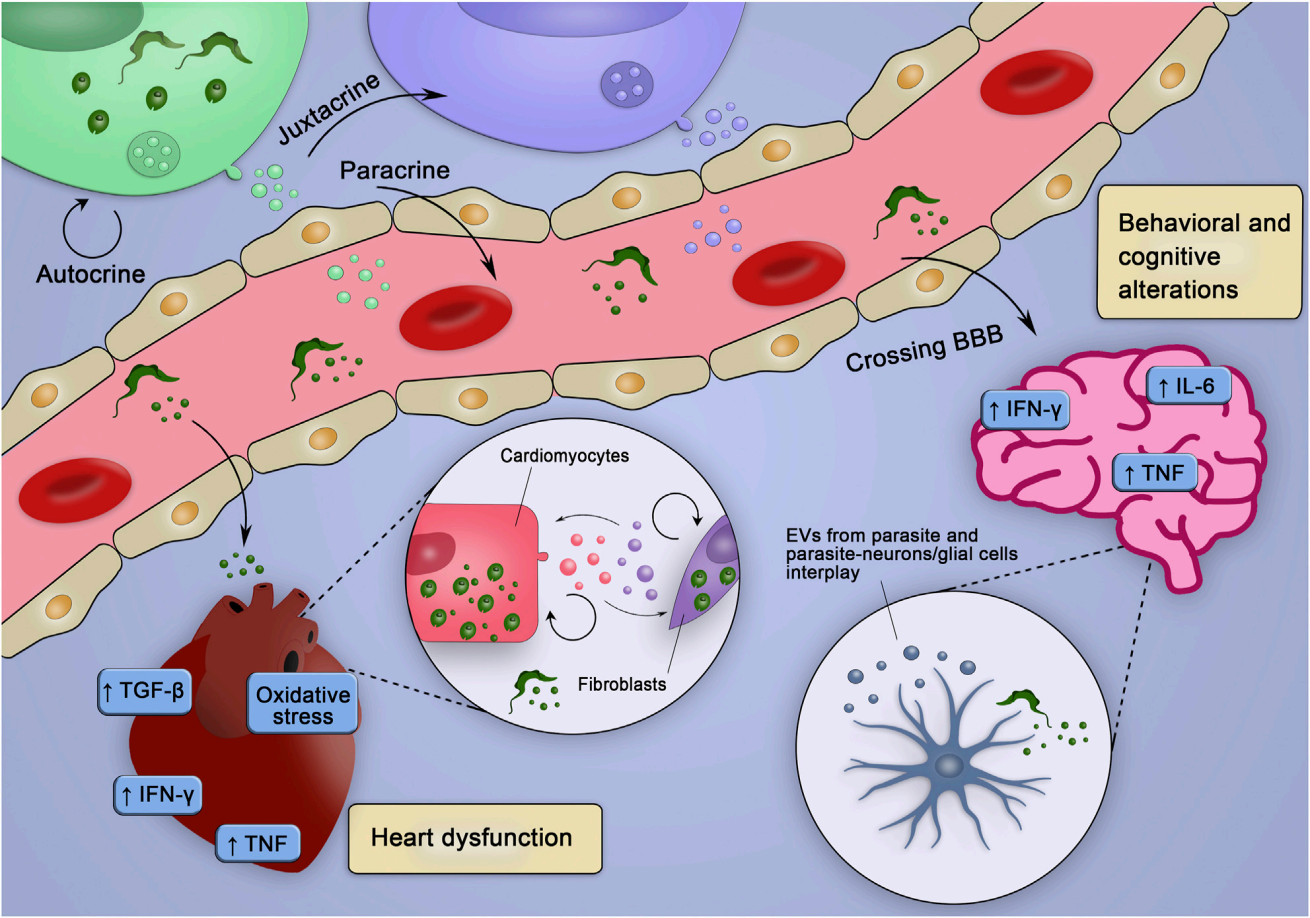
Hypothetical integrative view of the effects of various sources of EV in the pathogenesis of CD. EVs of distinct origins may act in an autocrine, juxtracrine, and/or paracrine way, contributing to create inflammatory milieu (enriched in inflammatory cytokines and mediators), which may trigger tissue-specific injury mechanisms leading to heart dysfunction and behavioral and cognitive alterations. EVs from parasite and infected cells could cross the blood-brain barrier (BBB), interact with neurons/glial cells and stimulate the production of cytokines and co-stimulatory molecules already related to behavioral and cognitive alterations. Alternatively, parasites could cross the BBB and infect glial cells, which could release EVs and affect surrounding cells. In the heart, EVs from the parasite could, at first, reduce the production of molecules that would fight the parasite and favor the establishment of the infection. Posteriorly, the EVs from infected cells could sustain the inflammatory profile observed during chronic infection contributing to the heart dysfunction.

EVs have been demonstrated to participate in the infection processes and chronic diseases, and these features are also applicable to CD. Thus, the present review discussed the potential role of EVs in remote signaling and promotion of inflammatory injury in CD, which is a chronic infectious disease with complex outcomes, considering the manifestations and severity of the clinical forms. EVs released in the acute phase of CD may favor parasite survival, evasion of complement-mediated lysis, and setting a regulatory immune response in the host cells, allowing parasite persistence. During the chronic phase, in turn, EVs induce a proinflammatory profile, upregulating inflammatory gene expression, and oxidative/nitrosative stress, thus potentially contributing to pathogenesis and disease progression. Increased EV levels in the serum, cerebrospinal fluid, and brain tissue correlate with cognitive impairment in HIV infection and AD, which are diseases related to neuroinflammation, which is characterized as the effects of peripheral inflammatory cells, i.e., leukocytes, in the CNS. Although neuroinflammation is absent in CD, it is tempting to speculate that observed psychiatric and neurocognitive alterations in chronic CD patients (anxiety, depression, and memory loss) may be related to EVs stimulated by *T. cruzi* interaction with the resident cells of the CNS or released by the parasite itself. The Ama forms of *T. cruzi* are mainly sheltered in astrocytes, and astrocyte invasion is favored by microglia-derived IFN-γ and autocrine TNF production by astrocytes (Silva et al., 2015; Silva et al., 2017). Thus, since EVs tend to favor an inflammatory profile (Robbins et al., 2016), potential participation of EVs released by the cells of the CNS in behavioral and cognitive changes in CD (Vilar-Pereira et al., 2012; Vilar-Pereira et al., 2021) deserves to be evaluated. Importantly, CHF is associated with the loss of myocardial function, hypertrophy, and fibrosis (Kemp and Conte, 2012), which are the features common with the cardiac manifestations of chronic CD. CARD CD patients with CHF have circulating EVs with inflammatory cargo and a potential to induce a proinflammatory profile. Furthermore, EVs from infected cells stimulate the proinflammatory PARP-1-cGAS-NF-κB pathway and may play a role in fibrosis in the heart tissue, since *T. cruzi* infection promotes a profibrotic response in the myocardium and macrophages via the PARP-1/AP-1 pathway (Choudhuri and Garg, 2020b). Therefore, one can speculate that EVs shed by the parasites or from infected fibroblasts/cardiomyocytes may sustain a proinflammatory milieu and the renin-angiotensin system, favoring cardiac fibrosis and contributing to the progression of the cardiac form of CD (Laugier et al., 2020; Farani et al., 2021). Additionally, it is reasonable to speculate that EVs from the intracellular parasite forms may act from the inside of the host cells and regulate the infection and its consequences. These general hypotheses involving the participation of EVs in injury of the CNS and heart tissue contributing to clinical outcomes are summarized in Figure 3. Importantly, our ambitious proposals are in agreement with a recent raised hypothesis that *T. cruzi*-shed EVs work as damage-associated molecular patterns recognized by host cells, which may activate NF-kB-driven inflammatory response (Pinge-Filho, 2021).

Notably, the present review does not intend to separate vesicle subtypes (small and large). However, conflicting data were reported in a few published studies, and possible differences promoted by subpopulations already identified in *T. cruzi* should be considered. *T. cruzi* has various secretion mechanisms, although no mechanistic studies on EV biogenesis in this parasite are available. *T. cruzi* presents various molecules previously described to be involved in EV formation in mammalian cells, suggesting that the parasite has potential machinery for the formation of small and large EVs. Small vesicles can be generated due to the participation of clathrin and clathrin-associated protein orthologs responsible for membrane trafficking in *T. cruzi* (Kalb et al., 2016). Additionally, the presence of inositol phosphoceramides, which function as GIPLs and glycoprotein lipid anchors in *T. cruzi*, has been described (De Lederkremer et al., 2011). N-Glycosylation has been shown to participate in EV release by melanoma cells and can play a part in EV release by the *T. cruzi* parasite forms, since Trypo presents increased level of protein glycosylation in parasite EVs associated with host cell invasion (Bayer-Santos et al., 2013; Alves et al., 2017). The presence of the small GTPase Rab protein homologs associated with TS trafficking to the membrane has been detected within EVs (Bayer-Santos et al., 2013) and in *T. cruzi* (Araripe et al., 2005; Niyogi and Docampo, 2015). Finally, the SNARE complex composition in trypanosomatids has been predicted (Murungi et al., 2014). Large vesicles, in turn, can originate due to participation of the lipid rafts, which are one of the cargo-sorting pathways (van Niel et al., 2018) associated with GPI-anchored molecules identified in *T. cruzi* EVs (Bayer-Santos et al., 2013; Mucci et al., 2017). The Rho GTPase homolog TcRho1 is expressed in the parasites, controls substrate adhesion, and is responsible for *T. cruzi* differentiation to trypomastigotes during its life cycle (De Melo et al., 2006). ARF1 protein homolog (TcArf1) was also described in all life forms of *T. cruzi* and may play a part in vesicle release (de Sá-Freire et al., 2003). Therefore, considerable information on biogenesis of *T. cruzi* EVs remains to be unveiled.

Lipids are the major component of EVs, and various enriched lipid classes may vary depending on EV sources. Furthermore, a correlation of EV lipid composition and the progression of chronic diseases has been discussed (Donoso‐Quezada et al., 2021). The lipid composition of EVs released by *T. cruzi* has not been investigated; therefore, some questions can be asked. Do lipids play a part in biogenesis of *T. cruzi* EVs? Do EV lipids modulate the host cell response to *T. cruzi* infection? Furthermore, a complete analysis of the cargo of EVs shed from the parasite and generated from the parasite-host interplay with regard to lipids, proteins, and nucleic acids (tRNA, miRNA, and DNA) may contribute to comprehension of interkingdom relationships and contribution of this communication tool to the key points of CD pathogenesis. As mentioned in the present review, glycans are present in EVs and may be involved in cargo recruitment and EV biogenesis (Williams et al., 2018), which is a matter that requires further studies of *T. cruzi-*host interplay. Competitive inhibitors or antibodies to glycans reduce the uptake of EVs by HeLa cells (Batista et al., 2011), suggesting the participation of glycans in the cell-EV interactions. *T. cruzi* glycoproteins have already been identified in EVs, such as TS family members and MASP family proteins. Various parasite strains release EVs containing various proteins and carbohydrates, and the same parasite form releases various EV populations with variable cargo. Thus, can the differences in glycosylation of a pool of EVs influence the biological response induced by a contact with the parasite itself or the target host cells? Should various subpopulations of EV, putatively containing various components, be studied in combination? Do distinct methods of analysis of glycans (Macedo-da-Silva et al., 2021) generate a bias regarding EV composition? These and other questions concerning EV composition and biological consequences triggered by EV cargo in the parasite-host cells (vertebrates or invertebrates) may evolve in the coming years.

At present, the underlying factors proposed to explain CD pathogenesis include 1) parasite persistence in the chronic phase, 2) unbalanced immune response associated with severity and disease progression, and 3) intrinsic factors in the tissues and organs (such as mitochondrial damage and oxidative stress). Thus, we have to learn considerable amount of information about the participation of EVs shed by the parasite forms, host-parasite interplay, and uninfected host cells exposed to inflammatory milieu in the tissues targets of parasite persistence and host reaction. Therefore, this review aims to encourage new researchers to embrace this challenging field.
